# Development of a Superhydrophobic Protection Mechanism and Coating Materials for Cement Concrete Surfaces

**DOI:** 10.3390/ma17174390

**Published:** 2024-09-05

**Authors:** Zihao Zhao, Shuai Qi, Zhi Suo, Tao Hu, Jiaheng Hu, Tiezheng Liu, Mengyang Gong

**Affiliations:** School of Civil and Transportation Engineering, Beijing University of Civil Engineering and Architecture, Beijing 100044, China; zihaozhao@126.com (Z.Z.); mmiwm123@163.com (T.H.); 15327346020@163.com (J.H.); 2108590023117@stu.bucea.edu.cn (T.L.); gongmengyang0209@163.com (M.G.)

**Keywords:** micro-nano rough structure, modified epoxy resin, superhydrophobic coating, contact angle, cement concrete

## Abstract

In order to further enhance the erosion resistance of cement concrete pavement materials, this study constructed an apparent rough hydrophobic structure layer by spraying a micro-nano substrate coating on the surface layer of the cement concrete pavement. This was followed by a secondary spray of a hydroxy-silicone oil-modified epoxy resin and a low surface energy-modified substance paste, which combine to form a superhydrophobic coating. The hydrophobic mechanism of the coating was then analysed. Firstly, the effects of different types and ratios of micro-nano substrates on the apparent morphology and hydrophobic performance of the rough structure layer were explored through contact angle testing and scanning electron microscopy (SEM). Subsequently, Fourier transform infrared spectroscopy and permeation gel chromatography were employed to ascertain the optimal modification ratio, temperature, and reaction mechanism of hydroxy-silicone oil with E51 type epoxy resin. Additionally, the mechanical properties of the modified epoxy resin-low surface energy-modified substance paste were evaluated through tensile tests. Finally, the erosion resistance of the superhydrophobic coating was tested under a range of conditions, including acidic, alkaline, de-icer, UV ageing, freeze-thaw cycles and wet wheel wear. The results demonstrate that relying solely on the rough structure of the concrete surface makes it challenging to achieve superhydrophobic performance. A rough structure layer constructed with diamond micropowder and hydrophobic nano-silica is less prone to cracking and can form more “air chamber” structures on the surface, with better wear resistance and hydrophobic performance. The ring-opening reaction products that occur during the preparation of modified epoxy resin will severely affect its mechanical strength after curing. Controlling the reaction temperature and reactant ratio can effectively push the modification reaction of epoxy resin through dehydration condensation, which produces more grafted polymer. It is noteworthy that the grafted polymer content is positively correlated with the hydrophobicity of the modified epoxy resin. The superhydrophobic coating exhibited enhanced erosion resistance (based on hydrochloric acid), UV ageing resistance, abrasion resistance, and freeze-thaw damage resistance to de-icers by 19.41%, 18.36%, 43.17% and 87.47%, respectively, in comparison to the conventional silane-based surface treatment.

## 1. Introduction

Cement concrete has become the most dominant material in structures such as airport runways and aprons because of its high strength after moulding and hardening and its ability to adapt to the environment [1]. In order to ensure the safety of aircraft landing and take-off, de-icing fluid is used to remove snow and ice from concrete pavements at airports in winter. However, this also causes more damage to the cement concrete road surface. This is due to the fact that the particles of cementitious materials are not tightly arranged during the curing process, and erosion media can easily penetrate into the interior of the material matrix through the capillary effect, resulting in accelerated destruction of the material by external water and erosion ions [2]. Studies have shown that ordinary cement concrete road surfaces, under the influence of de-icers, generally suffer from durability damage problems such as skinning, spalling, and stone exposure after 3 to 5 years [3,4]. Commonly used de-icers for cement concrete on airport road surfaces can be divided into two categories, one being organic alcohol-based aircraft de-icing fluids (AD), such as ethylene glycol (EG) or propylene glycol (PG). The other category is salt-based airport pavement de-icers (APD), mainly calcium magnesium acetate (CMA), potassium acetate (KAc), and sodium acetate (NaAc). In addition, it was found that cement concrete with a water-cement ratio of 0.42 to 0.64 showed surface spalling after 50 environmental freeze-thaw cycles of an organo-alcohol-based de-icing fluid. Bai et al. found that low-concentration calcium-magnesium acetate (a salt aircraft de-icer) de-icing solution caused freeze-thaw damage to concrete, while at higher concentrations it instead mitigated the occurrence of freeze-thaw damage to concrete [5]. Studies by Liu et al. and Chen et al. further pointed out that the degree of freeze-thaw cycle damage of cement concrete by de-icer solution is related to the de-icer solution’s own icing expansion rate, the concrete’s inhalation rate, and the inhalation rate of de-icer solution [6,7]. Therefore, preventing the absorption of solution by cement concrete is one of the keys to preventing erosion and freeze-thaw damage.

Various methods exist to improve the resistance of cement concrete to penetration of solution ions, among which surface coating is one of the effective methods. Superhydrophobic cementitious coatings are a type of protective coating for cement concrete. (It is generally accepted that static contact angle measurements >90°are considered hydrophobic, while static contact angle measurements >150°are considered superhydrophobic [8]). It achieves hydrophobicity by spraying a superhydrophobic modified material on the cementitious surface, which makes water less likely to adhere to the surface and removes it quickly. At the same time the coating applied to the concrete surface acts as a barrier, shielding the structure from free state ions and preventing the erosion of the concrete or its internal reinforcement, thus improving the durability of the cementitious material [9,10]. Zhao et al. prepared a superhydrophobic coating by the green sol-gel method for cementitious structures using nanosized SiO_2_, n-octyltriethoxysilane, and silane coupling agent, a commonly used low-surface-energy substance, which showed good chemical stability and resistance to UV irradiation [11]. Seongh et al. investigated the chloride ion permeation resistance of cement mortar specimens coated with an amine-based carbon dioxide (CO_2_) solvent and found that solutions with higher concentrations of monoethanolamine (MEA) captured more CO_2_ and, at the same time, could improve chloride ion permeation resistance by reducing the number of capillary macropores within the cement mortar specimens [12]. Sun et al. found that an appropriate amount of slag powder and iron tailing powder can make the internal structure of the coating more dense, which can reduce the surface energy, effectively improving its softening coefficient and enhancing the water resistance [13]. Xu et al. prepared a hydrophobic coating for cement mortar by adding stearic acid-modified superhydrophobic zeolite powder to reduce the surface energy, which significantly reduced the capillary water absorption of the mortar and improved the anti-erosion properties of the mortar [14]. The above studies illustrate that by adding low-surface-energy compounds to the coating substance, it helps to enhance the hydrophobic protection of the coating [15,16]. Yuan et al. prepared a superhydrophobic aluminium surface using femtosecond laser ablation and heat treatment, and the experimentally produced samples possessed excellent self-healing and anticorrosive properties [17]. Song et al. developed a fluorine-free superhydrophobic cement concrete surface coating, which had high surface mechanical strength and remained superhydrophobic after multiple scrapings or sandpaper abrasion for 20 m and still remained superhydrophobic [18]. In contrast, Liu et al. used nano-cast moulding technology to fabricate a microstructure on the concrete surface to prepare hydrophobic coatings [19]. Arabzadeh et al. used layer-by-layer jet deposition technique to prepare nanocoatings on concrete surface [20]. Wang et al. replicated the micro- and nano-dendritic structures of superhydrophobic copper mesh onto fresh concrete, leading to the formation of hierarchical structures on the surface of the concrete and further preparation of superhydrophobic coatings [21]. These studies show that low surface energy and the roughness of micro- and nano-structures are two factors worth considering when preparing superhydrophobic surfaces, which all can reduce the adhesion between contaminants and coatings and endow these surfaces with excellent superhydrophobicity. Therefore, the combination of micro-and nano-structures with low-surface-energy materials enhances the hydrophobicity of concrete surfaces.

Table 1 shows the technologies and materials used in common superhydrophobic protective coatings. A large number of superhydrophobic protective coating materials have been developed and are being used in a number of expanding applications, such as erosion protection and noise protection. The research and application of superhydrophobic coating materials have effectively improved the erosion and freeze-thaw resistance of cement concrete materials. Environmental factors such as UV irradiation and mechanical abrasion can cause functional degradation of the coating, affecting its effective service life [22]. In comparison to conventional cement concrete structures, roadway concrete is subject to environmental factors as well as direct vehicle/aircraft loads and wheel load abrasion, which places additional demands on the protective coating of roadway concrete beyond the resistance to environmental influences. It is worth noting that coating materials also have many kinds of functional roles, such as reducing the noise of the road surface due to the movement of traffic vehicles or antioxidant and corrosion protection for special structures. However, our research has focused more on improving the hydrophobicity of concrete pavement coatings. In this study, we firstly constructed a rough structure on the cement concrete road surface by using micron and nanoparticle substrates and sprayed a modified epoxy resin-low-surface-energy modifier slurry to form a wear-resistant and erosion-resistant cement concrete superhydrophobic protective coating (SPC) on the road surface. The study first tests the effect of different micro- and nano-substrate types and ratios on the apparent morphology and hydrophobic properties of rough structural layers. Subsequently, the optimum ratio of reactants, temperature, and reaction mechanism for epoxy modification were analysed, and the mechanical properties of the modified epoxy-low-surface-energy modifier pastes were verified. Finally, we verified the excellent erosion resistance of the superhydrophobic coating under acid, alkali, snowmelt, UV ageing, and freeze-thaw cycling conditions, and the effective anti-slip properties of the superhydrophobic protective coating through wet wheel abrasion tests and sanding tests.

## 2. Materials and Tests

### 2.1. Materials

#### 2.1.1. Substrates for Micro- and Nano-Rough Structured Layers

Combining micro- and nano-roughness with low-surface-energy materials can enhance the hydrophobicity of concrete surfaces [30]. In addition, it is difficult to achieve the superhydrophobic properties by relying on the original structure of the cement concrete surface, a conclusion that will be confirmed in Section 3.1. In this study, three-micron substrates of diamond micropowder, silicon carbide, and white corundum with nano-silicon dioxide substrate were selected to construct micro- and nano-rough structural layers on the surface of cement concrete.

**Figure 1:**
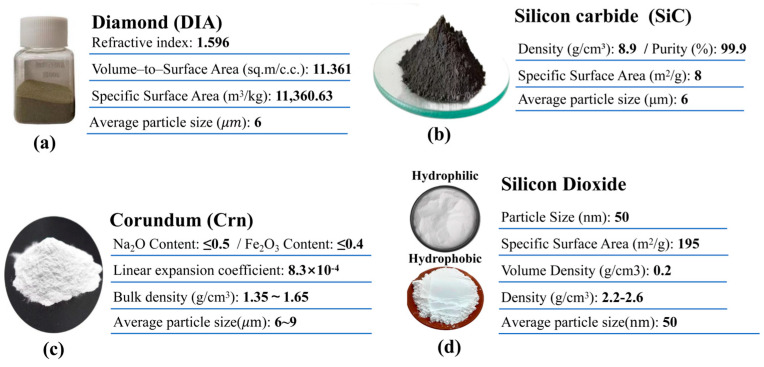
Micro/nano-rough structure layer substrates.

The conventional physical properties of each material are shown in Figure 1.

#### 2.1.2. Modified Epoxy Resins and Low Surface Energy Modifiers

Adding a low-surface-energy modifier is an important way to improve the hydrophobicity of coating materials, and polytetrafluoroethylene (PTFE) was chosen as the low-surface-energy modifier in this study. Meanwhile, E51 epoxy resin (E51) and curing agent were selected as the main binder of the coating, which together with anhydrous ethanol formed a solvent carrier for low-surface-energy modifiers and micro/nano-materials. In order to further improve the wear resistance of the coating, this study used hydroxyglycerol as a modifier and anhydrous magnesium sulphate and dibutyltin diacetate as a catalyst to modify the epoxy resin and prepare a modified epoxy resin with high strength and low surface energy.

**Figure 2:**
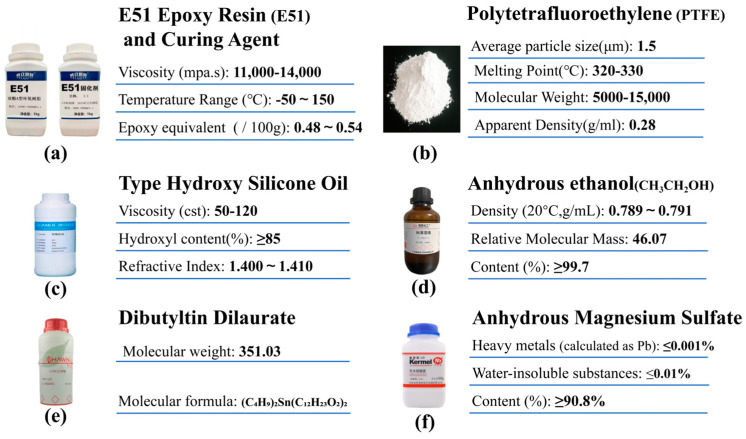
Bonding materials and modifiers.

The basic physical properties of each material are shown in Figure 2.

#### 2.1.3. Cement Concrete Materials

1. Cement and water reducing agent

P.O 42.5 type ordinary silicate cement was used, and its physical and mechanical properties are shown in Table 2.

2. Aggregate

In this study, standard sand (fineness modulus of 2.86) was used for fine aggregate and basalt-crushed stone was used for coarse aggregate, which were all provided by the Beijing Building Material Group (Beijing, China), and the technical indexes meet the specification MH 5006-2015. The design of synthetic grading was carried out by two kinds of aggregates from 5 to 10 mm and 10 to 20 mm. The range of grading and the basic properties of concrete satisfy the provisions of specification MH 5006-2015 [31].

#### 2.1.4. Other Materials Used in the Test

**Figure 3:**
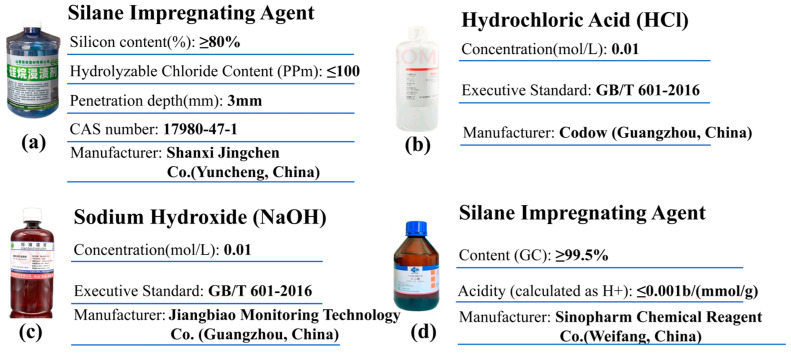
Other materials used in the experiment [32,33].

A silane surface treatment agent (SIA) was used as a control group to verify the actual hydrophobic properties of the superhydrophobic protective coating (SPC) produced. Hydrochloric acid, sodium hydroxide, and ethylene glycol were also used to simulate acidic, alkaline, and snowmelt environments to verify the erosion resistance of the SPC in this study. The basic indexes of each reagent are shown in Figure 3.

### 2.2. Tests

#### 2.2.1. Hydrophobicity Test

For the hydrophobicity study of the coating material, a video optical contact angle meter (OCA 50 AF, Dataphysics) was used to measure the contact angle (CA) of the sample surface at room temperature. More than three test spots were randomly taken from each sample surface, and the average value was taken as the final test result, with droplet volumes of 3 μL for CA tests [34,35].

#### 2.2.2. Functional Group and Molecular Weight Test

1. Fourier transform infrared spectroscopy (FTIR)

This technique was used in order to investigate the optimal ratio and reaction temperature conditions for the modification of epoxy resin by hydroxyl silicone oil and analyse the mechanism of the modification. In this study, a Bruker ALPHA model Fourier Transform Infrared Absorption Spectrometer (Houston, TX, USA) was used to test the functional group composition of the modified epoxy resin.

2. Gel permeation chromatography (GPC)

The molecular weight and molecular weight distribution of the modified epoxy resin were tested by gel permeation chromatography (GPC) using an Agilent 1260, with the detector Refractive Index, a flow rate of 1 mL/min, and a testing temperature of 35 °C.

#### 2.2.3. Micro-Morphological Testing

In order to explain the hydrophobic mechanism of the coating material from the micro-morphology point of view, the micro-morphology of the coating surface was observed and analysed using a Hitachi SU series scanning electron microscope (SU5000) in this study.

#### 2.2.4. Mechanical and Durability Tests

1. Slip resistance

The apparent roughness of the cement concrete after spraying the superhydrophobic protective layer was determined to test its slip resistance. This was conducted in accordance with the implementation of the relevant provisions of the hand-laying sand method in the MH/T 5110-2015 [36].

2. Abrasion resistance

The abrasion test was carried out on the coating material using an abrasion instrument (rotational speed of the abrasion head was 140 r/min ± 2 r/min and 61 r/min ± 1 r/min). Firstly, the concrete specimen coated with the SPC was placed in a tray, then the specimen was fixed with a jig, the test bench was lifted so that the abrasive head was in contact with the specimen and locked, and then the power supply was activated for the test. In this study, the rotation of the abrasive head by 1 r was defined as the abrasion of the abrasive head against the surface of the specimen for one time, and the change rule of the size of the contact angle was used to characterise the abrasion resistance of the coating.

3. Tensile test

In this study, modified epoxy resins with low surface energy were prepared and subjected to tensile fracture tests. The tests were carried out using an MTS electronic universal testing machine with a tensile speed of 10 mm/min to obtain the tensile strength and modulus of elasticity as the indexes for evaluating its mechanical properties.

4. Erosion resistance test

**Figure 4:**
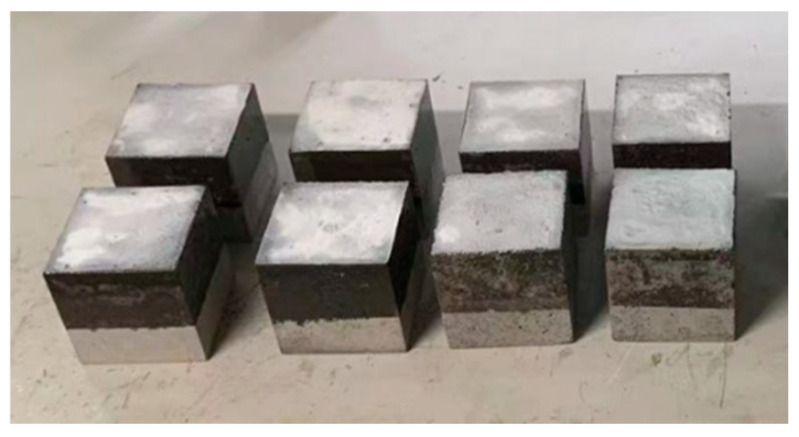
Preparation of specimens (side sealing method).

In order to test the erosion resistance of the SPC, a solution of hydrochloric acid with a pH of 3, a solution of sodium hydroxide with a pH of 10, and a solution of ethylene glycol with a concentration of 3% were prepared for use in this study. These solutions were used to simulate the erosion of the superhydrophobic protective layer of cement concrete pavements by acids, alkalis, and de-icers. At 5 h intervals throughout the erosion test, the specimens were removed from the corrosive solution and cleaned with distilled water. They were then left to stand at room temperature for 30 min to dry. Subsequently, the static contact angle of water droplets was measured for each group of specimens, and the average value was calculated in order to analyse the erosion resistance of the superhydrophobic coatings. As shown in Figure 4, it should be noted that in this test, only the SPC-coated side of the cement concrete specimen was immersed in the corrosive liquid, and the other side of the specimen was sealed and isolated with epoxy resin in order to prevent erosion on the side of the specimen from affecting the test results on the coated surface.

5. UV ageing resistance test

In this study, a UV lamp with an intensity of 70 uW/cm^2^ was used to simulate outdoor UV illumination to conduct UV aging of the coating for a maximum of 20 d. The contact angle and rolling angle of the coating surface were measured every 1 d to investigate the effect of UV on the hydrophobic properties of the SPC [34,35].

6. Resistance to de-icing fluid and freeze-thaw cycle damage test

In order to further test the freeze-thaw damage resistance of the SPC, this study refered to the requirements of GB/T 50082-2009 [31], and adopted 100 mm × 100 mm × 100 mm moulds to prepare cement concrete specimens. In order to comply with the stipulation that the test area should be no smaller than 0.08 m^2^, a minimum of eight specimens were prepared for each group, and the specimens were coated with the SPC. In accordance with MH 5006-2015, the freeze-thaw damage test method for cement concrete resistance to de-icing fluid was employed [37]. This solution was prepared with a mass ratio of 97% distilled water and 3% ethylene glycol and was used as the test erosion solution. A total of 30 freeze-thaw cycles were conducted, with the amount of freeze-thaw stripping per unit surface area of cement concrete calculated after every five cycles. The silane surface treatment was also tested as a control for the SPC.

### 2.3. Specimen Preparation Method

In this study, it is recommended to first spray the micro/nano-rough structure coating on the cement concrete surface, followed by spraying the modified epoxy resin-PTFE (low-surface-energy modifier) slurry to make the superhydrophobic protective coating [34,35]. The preparation process of the superhydrophobic protective coating (SPC) and the ratio of each material will be given a detailed description in Section 3.3.

## 3. Results and Discussion

### 3.1. Micro- and Nano-Rough Structure Coatings

**Figure 5:**
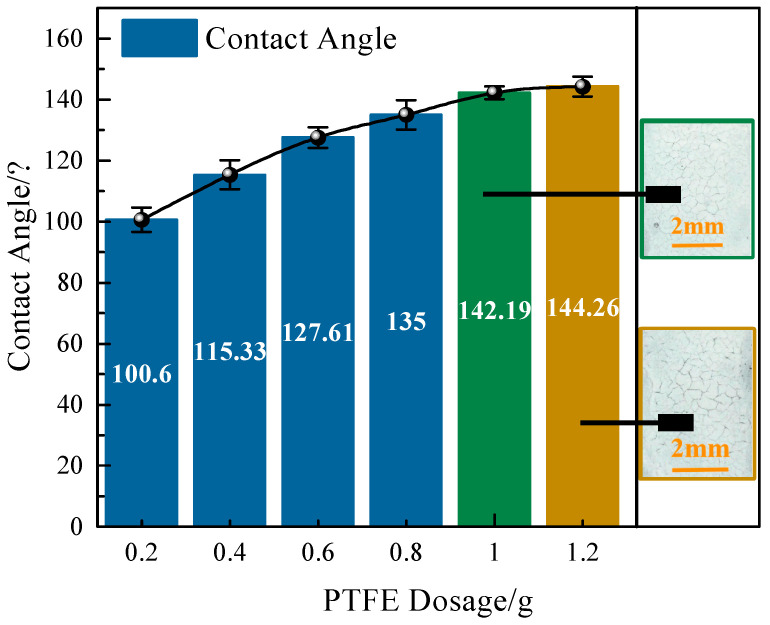
Influence of concrete’s inherent coarse structure under various preparation processes on the hydrophobicity of PTFE coatings at different dosages of PTFE.

Firstly, the authors only formulated the coating materials using different ratios (1:1, 1:2, 1:3, 1:4, 1:5 and 1:6) of low-surface-energy modifiers and unmodified epoxy resin at the initial stage of the study (0.2 g of epoxy resin was used in this case), and the results of the contact angle measurements are shown in Figure 5. 

As can be seen from Figure 5, the maximum value of the contact angle of the coating surface at several ratios is less than 150°, which indicates that it is difficult to achieve superhydrophobicity performance solely by relying on the rough structure of the concrete surface. Therefore, constructing micro- and nano-scrubby structural layers on the cement concrete surface to enhance the superhydrophobicity performance of the coating was considered [34]. In addition, the contact angle values increase with increasing PTFE doping, but the slope of the contact angle growth decreases gradually. This suggests that the addition of more PTFE powder may not have further improved the superhydrophobic properties of the coating. It is worth noting that when too much PTFE is used, the coating cracks significantly after curing, as shown in Figure 5.

#### 3.1.1. The Magnitude of the Effect of Substrate Ratio on the Hydrophobicity of Micro- and Nano-Rough Structure Coatings

**Figure 6:**
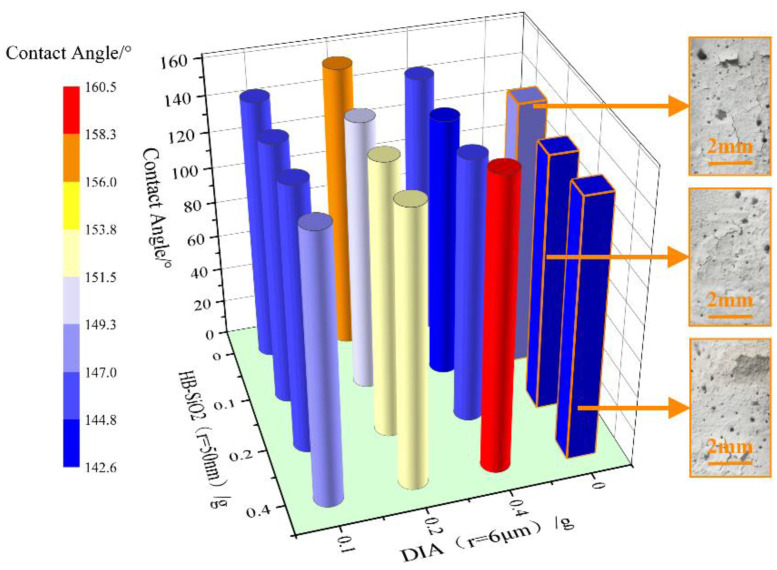
Contact angle measurements under different ratios of micro- and nano-rough structure coatings.

In this study, the epoxy resin (0.2 g)/PTFE (0.8 g) mass ratio was set to 1:4 to explore the effect of different hydrophilic nano-silica (HB–SiO_2_, particle size of 50 nm) and DIA (particle size of 6 μm) ratios on the hydrophobicity of micro- and nano-rough structure coatings. The test results are shown in Figure 6.

As illustrated in Figure 6, the coatings lacking the addition of DIA exhibited reduced hydrophobicity and displayed pronounced cracking (see images of the coatings highlighted in orange in Figure 6). Upon mixing HB–SiO_2_ and DIA in the ratio of HB–SiO_2_: DIA = 1:1, the resulting coating exhibited the highest contact angle of 160.46° (see the cylindrical icon highlighted in red in Figure 6), indicative of excellent hydrophobicity. It was deduced that this was possibly due to the fact that when DIA was used in combination with HB–SiO_2_, the nanoscale HB–SiO_2_ particles were first attached to the surface of the DIA micropowder and subsequently combined with the concrete surface structure to form a complex roughness structure, which exhibited better hydrophobicity. It is worth noting in Figure 6 that when the DIA dosage is 0.1 g, the contact angle of the coating changes insignificantly with the increase of the HB–SiO_2_ dosage. However, when the DIA dosage was 0.4 g, the contact angle of the coating’s surface increased significantly with the increase of the HB–SiO_2_ dosage. This indicates that when the DIA dosage was higher, the HB–SiO_2_ dosage significantly affected the hydrophobicity of the coating.

#### 3.1.2. Influence of Material Types on the Hydrophobicity of Micro- and Nano-Rough Structure Coatings

**Figure 7:**
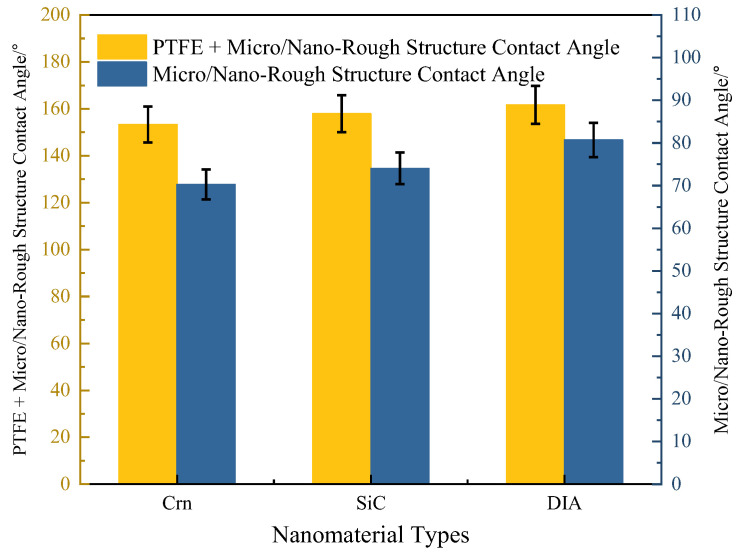
Surface contact angle magnitude of coatings under various microstructure types.

In order to compare the effects of different nanomaterial on the hydrophobicity of superhydrophobic coatings, diamond powder, silicon carbide powder, and white corundum powder with a particle size of 6 μm were used to form micro- and nano-rough structure coatings with HB–SiO_2_, and their hydrophobicity was tested. As illustrated in Figure 7, the utilisation of micro- and nano-structured layers in isolation, without the incorporation of PTFE, is inadequate to attain the desired hydrophobicity. However, when the three micro- and nano-structured layers of diamond micropowder, silicon carbide micropowder, and white corundum were combined with PTFE, the contact angle of the coating surface reached 153.27°, 157.94° and 160.46°, which can be regarded as possessing superhydrophobicity, and diamond micropowder had the best effect.

**Figure 8:**
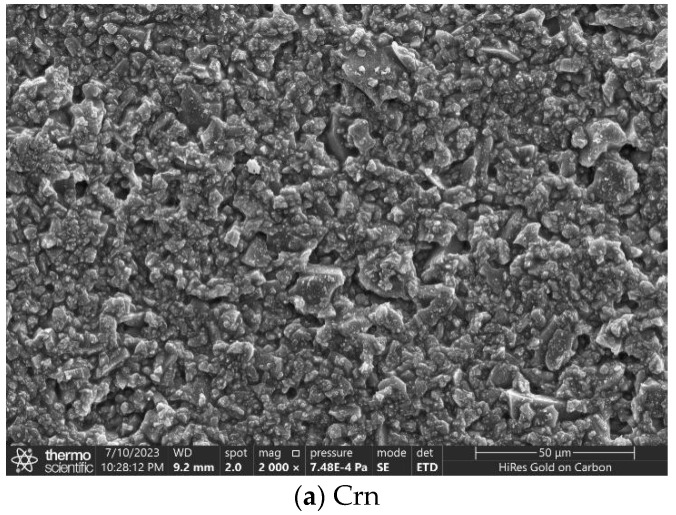

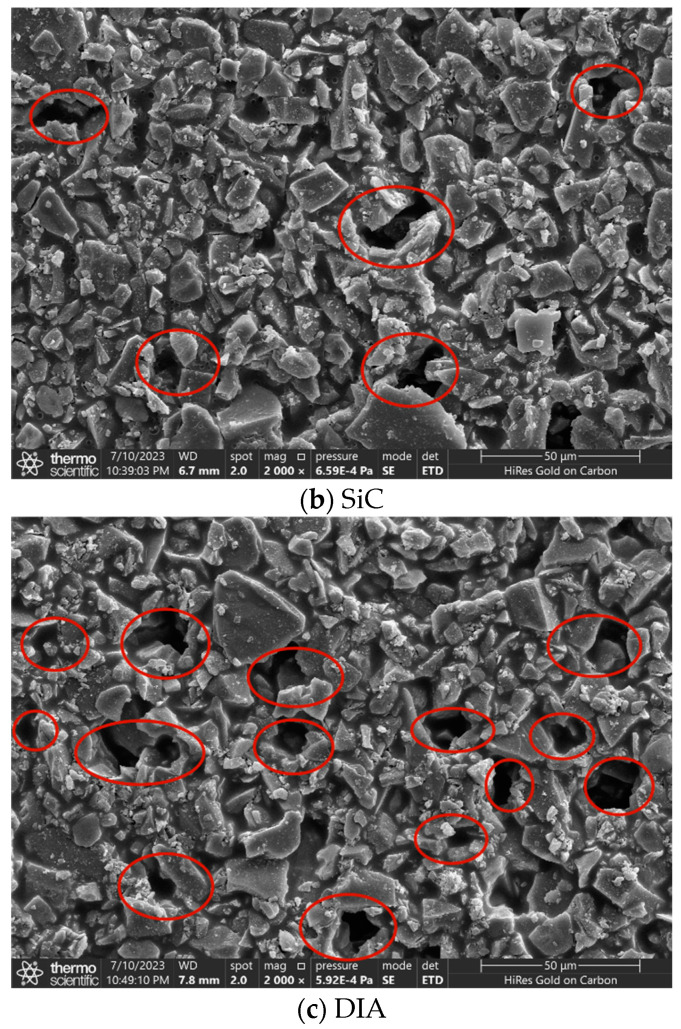
The microscopic morphology of surfaces with three types of micro- and nano-rough structure coatings.

The apparent morphology of the three micro- and nano-roughened coatings was further tested using scanning electron microscopy (SEM) and is shown in Figure 8. We can see that when using Crn, SiC, and DIA as micron structures, they all have a large number of HB–SiO_2_ nanoparticles attached to their surfaces. This stacking structure increases the roughness of the coating surface, so it makes the coating produce good hydrophobicity. Compared with Crn and SiC, DIA coatings have more “air chamber” structures on the surface, which help the coating (They are circled in red in Figure 7). surface capture more air and thus enhance the surface hydrophobicity. Therefore, the hydrophobicity of DIA micro- and nano-structured layers is better.

#### 3.1.3. Analysis of Micron Substrate Types on the Wear Resistance of Superhydrophobic Protective Coatings

As can be seen from Figure 6 in Section 3.1.1, when HB–SiO_2_ alone was used to construct rough structure coatings, cracks appeared in the coatings, which indicated that HB–SiO_2_ had a detrimental effect on the bonding stability between the coatings and the specimens. This problem was solved in this study by using hydrophilic silica (HL–SiO_2_) instead of HB–SiO_2_ to construct nanostructures. On this basis, micro- and nano-rough structure coatings (with the addition of PTFE) of white corundum, silicon carbide, and diamond were prepared for wear resistance tests. Meanwhile, in order to verify the effect of binder strength on the wear resistance of the coatings, a control experimental group without the addition of epoxy resin was set up for this part.

**Figure 9:**
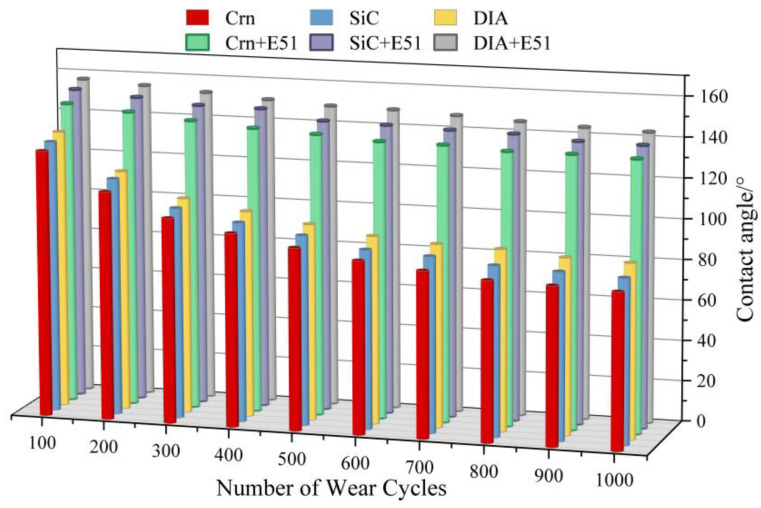
The variation pattern of contact angle with friction cycles.

As seen in Figure 9, the contact angle of the rough structure coatings decreased with the increase in the number of abrasions. The contact angle of the rough structure coatings without the addition of epoxy resin decreased by more than 33.86%, 34.16%, and 33.61% when the number of abrasions was only 300. This is because in order to ensure the hydrophobicity of the coating material, the PTFE material needs to be placed in the surface layer as much as possible, and the lack of binder leads to insufficient bonding of the PTFE powder to the substrate, resulting in large-scale detachment of the PTFE modification layer under the action of abrasion and a significant reduction in the hydrophobicity of the coating. This also illustrates that the strength of the binder in the coating material significantly affects the durability of the coating. Compared to the experimental group that lacked the epoxy resin as a carrier, the addition of the E51 epoxy resin resulted in significantly better abrasion resistance. This suggests that enhancing the bonding between low-surface-energy modifiers and micro- and nano-rough structure coatings is one of the effective measures that can be used to enhance the service life of superhydrophobic protective coatings [35]. Compared with corundum and silicon carbide, coatings prepared with diamond have stronger wear resistance.

In summary, the ratio of E51:HL–SiO_2_:DIA:AE = 1:2:2:2 was chosen to construct the rough structure coatings in this study. 

### 3.2. Low Surface Energy Modifier Pastes

From the analysis of Section 3.1, it can be seen that the enhancement of the bonding force between the low-surface-energy modifier and the micro- and nano-rough structural layer is a powerful measure to enhance the wear resistance of the superhydrophobic protective coating and guarantee its service life. In this study, the modified–E51 epoxy resin was prepared by adjusting the reaction conditions to promote the reaction between hydroxyl silicone oil and epoxy resin in the mode of dehydration condensation. This is because the dehydration condensation reaction can retain the epoxy group of the epoxy resin to a greater extent, and the epoxy group is the key functional group for the reaction between the epoxy resin and the curing agent. Its retention can enable the reaction between the epoxy resin and the curing agent to take place fully and enhance the comprehensive performance of the epoxy resin.

Preparation: ① Take E51 epoxy resin in a container and place it on the induction cooker for preheating for about 15 min. ② When the temperature of E51 epoxy resin is kept at the predetermined reaction temperature, weigh the hydroxyl silicone oil according to the corresponding proportion and add it into E51 epoxy resin slowly, stirring evenly. ③ Add dibutyltin diacetate (catalyst; dosage of 2–3% of hydroxyl silicone oil) and anhydrous magnesium sulphate (dehydrating agent; dosage of 2% of hydroxyl silicone oil) and then react at a constant temperature for 1.5–2 h to complete the modification. The proportions of the reactants will be given by the results of the studies in Section 3.2.1 and Section 3.2.2.

#### 3.2.1. Modification Mechanism of E51 Epoxy Resin

In order to obtain the optimal dehydration condensation reaction conditions of the epoxy resin and modifier, this study set the mass ratio of E51 epoxy resin (fixed 20 g) to hydroxyl silicone oil as 10:1, 8:1, 6:1, 4:1 and 2:1, and set three reaction temperatures of 130 °C, 140 °C, and 150 °C for pre-testing. (In this study a product prepared at a reaction temperature of 130 °C and the mass of E51 epoxy resin:mass of hydroxy-silicone oil (HSO) = 10:1 was recorded as 130 °C–10:1, and so on). 

**Figure 10:**
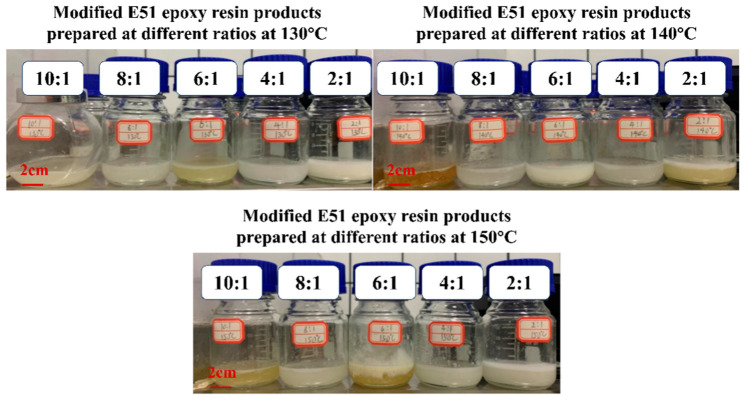
Products of hydrophobically modified E51 epoxy resin prepared at various temperatures and ratios.

Figure 10 illustrates the results of the pre-experiment, and it was seen that slight delamination occurred in the 130 °C–10:1, 130 °C–8:1, 140 °C–10:1, 140 °C–8:1, 150 °C–10:1, and 150 °C–8:1 experimental groups. Stratification was worse at 130 °C–6:1, 140 °C–4:1, 140 °C–2:1, 150 °C–6:1, 150 °C–4:1, and 150 °C–2:1. Discolouration occurred in the 130 °C–6:1, 140 °C–10:1, 140 °C–2:1, 150 °C–10:1, and 150 °C–6:1 experimental groups. Only 130 °C–4:1, 130 °C–2:1, and 140 °C–6:1 are stable milky-white viscous liquids, where no discolouration or delamination has occurred.

**Figure 11:**
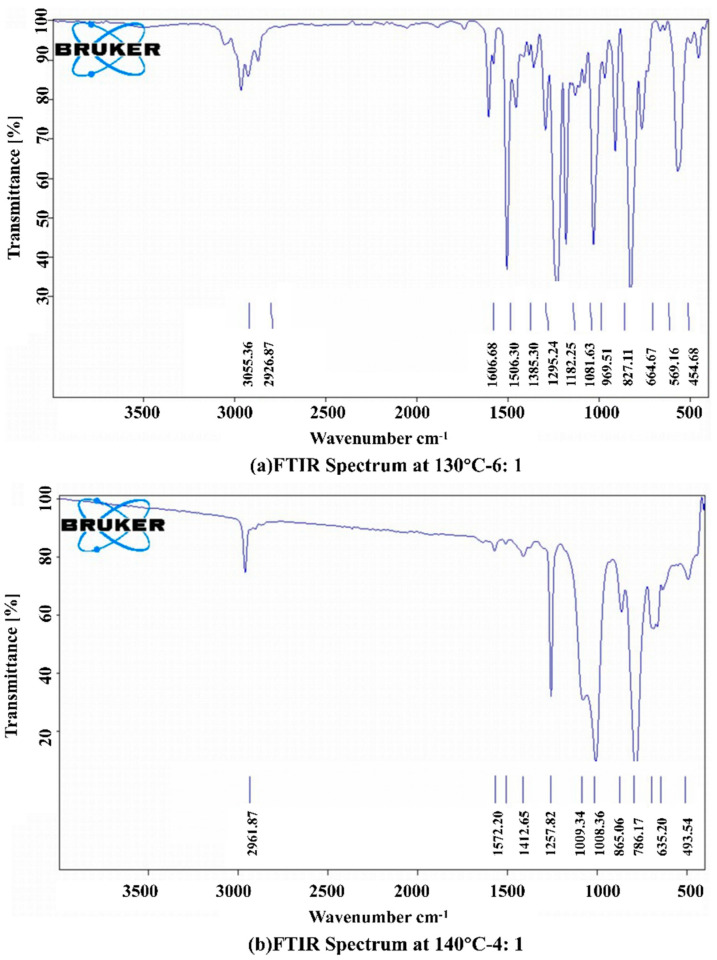
Infrared spectrum test results.

FTIR analyses were performed on the 130 °C–6:1, 130 °C–4:1, 130 °C–2:1, 140 °C–8:1, 140 °C–6:1, 140 °C–4:1, 150 °C–10:1, 150 °C–8:1, and 150 °C–6:1 experimental groups. Taking the infrared spectra of the reactants at a 6:1 mass ratio of E51 and hydroxyl silicone oil at 130 °C as an example, the absorption peaks of the C–C stretching vibration on the main chain at 1229.99 cm^−1^, 1182.25 cm^−1^, and 1131.53 cm^−1^ can be seen in Figure 11a. The in-plane bending vibrational absorption peak of Ar–H on the benzene ring at 1031.38 cm^−1^ and the asymmetric stretching vibrational absorption peak of C–O–C on the main chain at 1081.63 cm^−1^ can also be observed. The out-of-plane bending vibrational peak of C–H occurred at 969.51 cm^−1^. The telescopic vibrational absorption peak of epoxy group C–O–C occurred at 911.97 cm^−1^. Therefore, it can be judged that the product contains epoxy groups, which means that the hydroxyl silicone oil has undergone a dehydration condensation reaction with the E51 epoxy resin. Figure 11b shows the infrared spectrum of the reaction product at 140 °C–4:1. It can be seen that there is a bending vibrational absorption peak at 1257.82 cm^−1^ which was attributed to Si–CH_3_. The asymmetric stretching vibrational absorption peak at 1079.24 cm^−1^ was attributed to C–O–C. The stretching vibrational absorption peak at 1008.36 cm^−1^ was attributed to Si–O–Si on the main chain. The telescopic vibrational absorption peak at 865.06 cm^−1^ was attributed to Si–C on the main chain. The rocking vibrational absorption peak at 786.17 cm^−1^ was attributed to –CH3 [38]. No epoxy group was found in this reaction product, so it can be inferred that the epoxy ring-opening reaction between the hydroxyl silicone oil and the epoxy resin took place under the 140 °C–4:1 condition. 

The test results for the remaining reaction conditions are summarised in Table 3. 

As shown in Table 3, it can be seen that the higher the reaction temperature of the epoxy resin and hydroxyl silicone oil, the lower the probability of the epoxy ring-opening reaction. The test conditions that can be used for the dehydration condensation reaction are as follows: 130 °C–10:1, 130 °C–8:1, 130 °C–6:1, 130 °C–4:1, 140 °C–10:1, 140 °C–8:1, 140 °C–6:1, 150 °C–10:1, and 150 °C–8:1.

**Figure 12:**
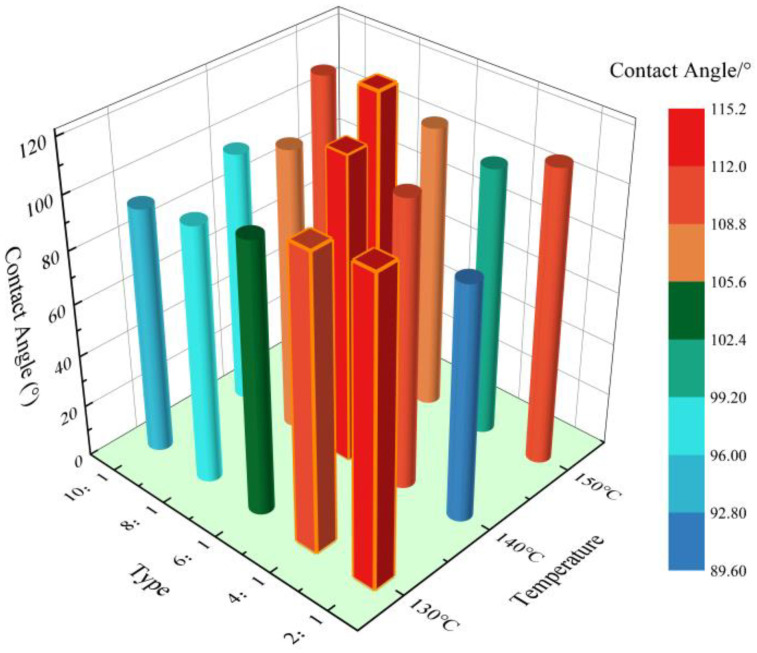
Contact angle measurement results of modified epoxy resin–PTFE slurry.

The modified epoxy resin was further prepared under the above conditions and PTFE was added to prepare modified epoxy resin–PTFE pastes to test their hydrophobic properties. The results are shown in Figure 12.

**Figure 13:**
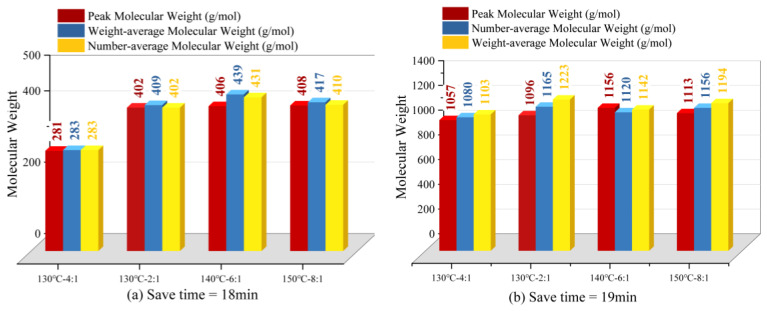
Penetration gel chromatography test results.

As shown in Figure 12, the hydrophobicities of the modified epoxy-polytetrafluoroethylene pastes under each reaction condition were ranked as 140 °C–6:1 > 130 °C–2:1 > 150 °C–8:1 > 130 °C–4:1 (see red prism markers in Figure 12), which was in agreement with the molecular weight ranking results of permeation gel chromatography, as shown in Figure 13. It can be seen from Figure 13 that the molecular weights of the modified epoxy resins were different under different reaction conditions. This is due to the dehydration condensation of the secondary hydroxyl group on the epoxy resin with the hydroxyl silicone oil, and the free small molecule hydroxyl silicone oil polymerises with the hydroxyl group at the end of the grafted siloxane chain on the epoxy resin at high temperatures to produce the grafted polymer [39,40]. The longer the grafted side chain, the larger the molecular weight of the grafted polymer and the better the modification effect on the epoxy resin [41].

Combined with the results of infrared spectroscopy and permeation gel chromatography, it can be found that the hydroxyl silicone oil and epoxy resin in this study will be accompanied by polymer polymerisation to form grafted polymers, and the content of grafted polymers is positively correlated with the hydrophobic properties of modified epoxy resin–PTFE pastes.

#### 3.2.2. Mechanical Property Test of Modified Epoxy Resin–PTFE Paste

**Figure 14:**
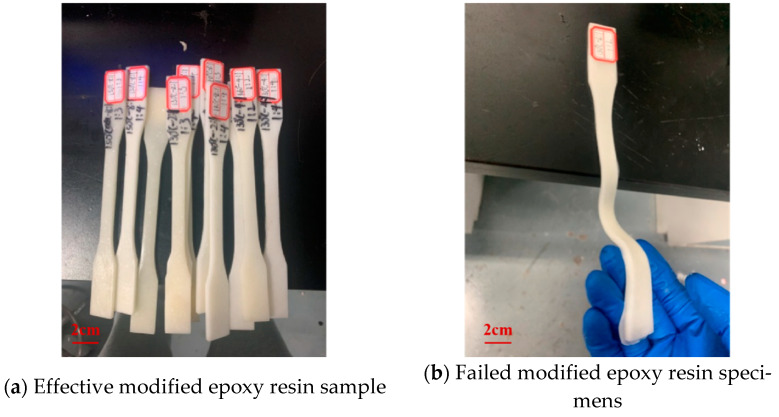
Tensile test specimens.

The modified epoxy resin was prepared at 140 °C–6:1, 130 °C–2:1, 150 °C–8:1, and 130 °C–4:1, and then PTFE was added to it in the ratios of 1:2, 1:3, and 1:4 to prepare epoxy–PTFE pastes. Finally, the epoxy–PTFE pastes were subjected to tensile tests. (In this study the product prepared at 140 °C–6:1 with modified E51 epoxy resin and PTFE powder at a mass ratio of 1:2 was defined as follows: 140 °C–6:1–1:2; and others by analogy). Among them, Figure 14 shows the modified epoxy resin–PTFE paste specimens. As seen in Figure 14b, the curing effect of the modified epoxy resin–PTFE paste at 130 °C–2:1–1:2 is poor, which is attributed to the excessive epoxy ring-opening reactions that occurred at 130 °C–2:1, and this specimen was discarded from the subsequent analysis.

**Figure 15:**
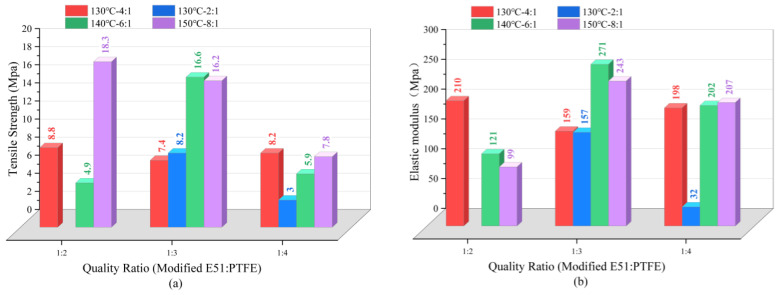
Tensile strength test results of modified epoxy resin–PTFE paste. (**a**) Tensile strength; (**b**) Elasticity Modulus.

As shown in Figure 15, the tensile strength of each type of modified epoxy resin–PTFE paste was 150 °C–8:1–1:2 > 140 °C–6:1–1:3 > 150 °C–8:1–1:3 > 130 °C–4:1–1:2. The elastic modulus relationship was 140 °C–6:1–1:3 > 150 °C–8:1–1:3 > 130 °C–4:1–1:2 > 150 °C–8:1–1:4. The tensile strength and modulus of elasticity of the modified epoxy–PTFE pastes at 140 °C and 150 °C showed a general pattern of increasing and then decreasing with the increase of PTFE mass ratio. This indicates that the PTFE powder has an enhancing effect on the tensile strength and elasticity of the modified E51 epoxy resin at 140 °C–6:1 with the proportion of the two in the appropriate amount, but with a further increase in the dosage, it affects the internal homogeneity of the material and thus leads to a decrease in the performance of the material. The 140 °C–6:1–1:3 showed the largest modulus of elasticity, and the tensile strength was second only to that of 150 °C–8:1–1:2. Moreover, in conjunction with the study in Section 3.2.1, that showed the hydrophobicity of 140 °C–6:1 modified epoxy resin is better, the 140 °C–6:1–1:3 conditions were chosen to prepare modified epoxy resin–PTFE paste in this study.

### 3.3. Durability Study of Superhydrophobic Protective Coatings

**Figure 16:**
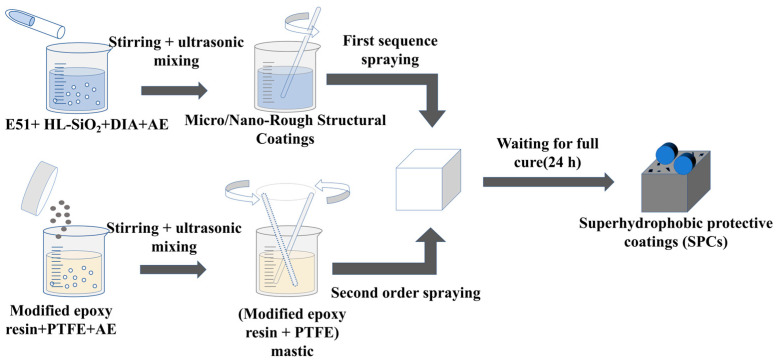
Process for the preparation of superhydrophobic protective coatings (SPCs).

Combining the results of Section 3.1 and Section 3.2, the process shown in Figure 16 was used in this study to prepare the superhydrophobic protective coating for cement concrete on the road surface and can be described as follows:

1. Mix the epoxy resin with anhydrous ethanol and stir for 1/4 h. Add the curing agent at the ratio of 1:1 with the epoxy resin and stir for 1/12 h. Add the micrometer-substrate and nano-substrate in turn, stir for about 1/6 h, and apply ultrasonic dispersion for 1/12 h. This results in the production of the micro- and nano-rough structure coatings (material ratio: E51:HL–SiO_2_:DIA:AE = 1:2:2:2).
2. Take an appropriate amount of modified epoxy resin with anhydrous ethanol and add PTFE (material ratio: modified–E51:PTFE:AE = 1:3:5.5) according to the provided proportion, stirring for about 1/6 h, and then apply ultrasonic dispersion for 1/12 h to make the modified epoxy resin–PTFE paste.
3. Spray the micro- and nano-rough structure coatings and modified epoxy resin–PTFE paste on the cement concrete surface in sequence. The superhydrophobic protective coating is formed after sufficient curing [34,35].

#### 3.3.1. Erosion Resistance

**Figure 17:**
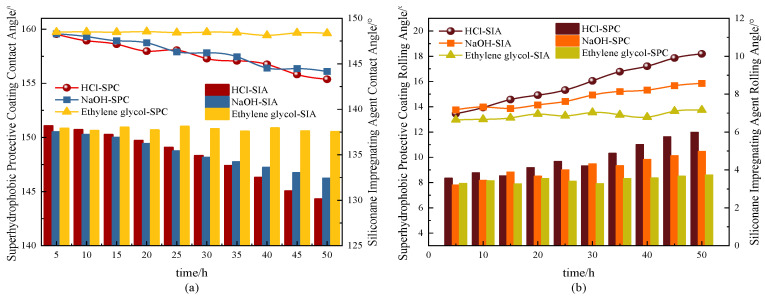
Contact angle and rolling angle test results. (**a**) Contact Angle; (**b**) Rolling Angle.

In this study, hydrochloric acid solution with pH = 3, NaOH solution with pH = 10, and ethylene glycol solution with a concentration of 3% were selected to conduct erosion resistance tests on superhydrophobic protective coating (SPC) and compared to the silane surface treatment agent (SIA). The erosion resistance of the coatings was analysed through the changes in hydrophobicity before and after the erosion tests. Figure 17 demonstrates the variation patterns of contact angle and roll angle of the coating surface with solution erosion time. As seen in Figure 17, the hydrophobicity of both coatings decreased after erosion by acid and alkali solutions. The surface contact angle of the superhydrophobic protective coating specimens treated with pH = 3 hydrochloric acid solution for 50 h decreased from 159.76° to 155.37°, which is a decrease of 2.75%, and the rolling angle increased from 3.11° to 5.99°, which is an elevation of 2.88°. The surface contact angle of the superhydrophobic protective coating treated with a pH = 10 sodium hydroxide solution for 50 h was reduced by 2.29% from 159.76° to 156.10°. The roll angle increased from 3.11° to 4.98°, an elevation of 1.87°, still maintaining strong hydrophobicity. Compared to SIA, the erosion resistance (in terms of HCl) of the SPC was improved by 19.41%. On the one hand, this is due to the fact that the Si–O bonds in the SIA react with hydrochloric acid solutions and sodium hydroxide solutions, leading to the hydrolysis and destruction of the film, thus reducing its surface hydrophobicity. On the other hand, the C–F bonds in the molecular structure of PTFE used in this study are highly resistant to both acid and alkali solutions and show better chemical stability on a macroscopic level, thus making the superhydrophobic protective coating more resistant to acid and alkali environments.

In addition, both the SPC and SIA showed strong erosion resistance to ethylene glycol solutions in this study. Subsequent freeze-thaw cycle testing of the coatings in ethylene glycol solution environments will be conducted to further validate their durability.

#### 3.3.2. UV Ageing Resistance

**Figure 18:**
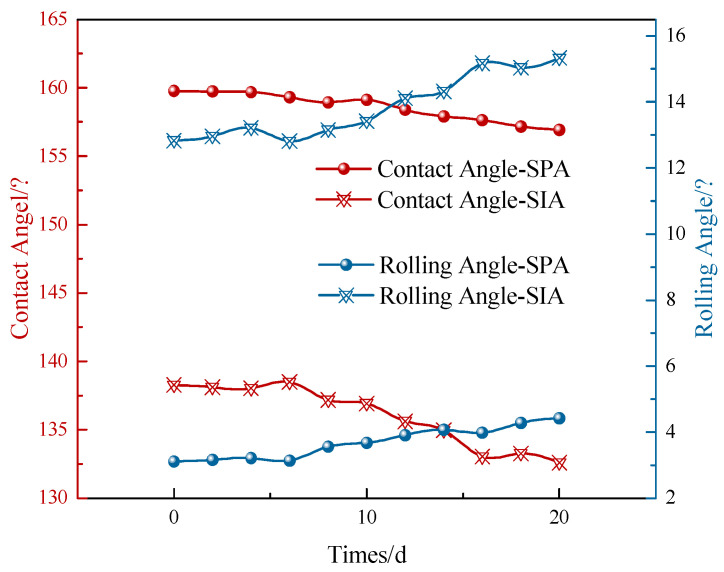
Variation of Contact Angle and Rolling Angle of Coating Surface with UV Irradiation Time.

After the SPC and SIA were sprayed on the surface of cement concrete specimens respectively, they were placed in a UV aging chamber for UV aging resistance test. Figure 18 shows the variation rule of contact angle and rolling angle of two protective coating surfaces with UV aging time. As seen in Figure 18, the surface contact angle of the SPC changed from 159.76° to 156.93° after 20 d of UV aging, which decreased by 1.77%. In addition, the rolling angle changed from 3.11° to 4.42°, which increased by 1.31°. The surface contact angle of the SIA changed from 138.27° to 132.59° after 20 d, which decreased by 4.11%, and the rolling angle changed from 12.83° to 15.33°, increasing by 2.5°. Compared to the SIA, the SPC has better UV ageing properties. This is due to the extremely high resistance to UV irradiation of the C–F bonds in the blends of PTFE and modified E51 epoxy resin. At the same time, the organic components in conventional silane impregnants, such as isooctyl and isobutyl, may be affected by photochemical reactions to occur chain breakage or structural rearrangement, resulting in changes in the molecular structure and weakening its chemical bonding ability with the cement concrete surface.

#### 3.3.3. Abrasion Resistance

**Figure 19:**
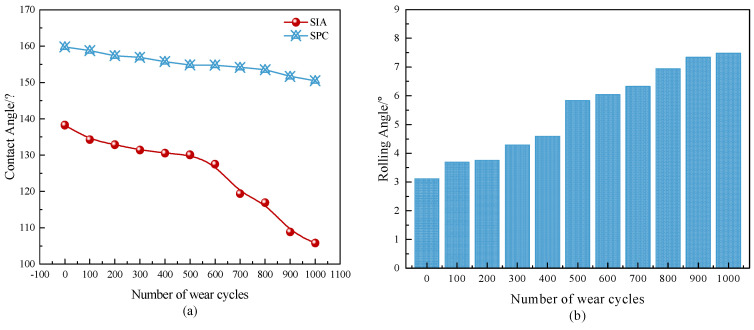
Variation of the coating surface contact angle with the number of abrasion cycles. (**a**) Contact Angle; (**b**) Rolling Angle.

As seen in Figure 19, the SPC surface contact angle decreases from 159.76° to 151.49°, a decrease of 5.18%, after undergoing 1000 abrasion cycles. In contrast, the surface contact angle of the SIA was reduced from 138.27° to 105.81° after 1000 abrasion cycles, a reduction of about 23.44%. Abrasion resistance is improved by 43.17% compared to the SIA. This is mainly due to the modified epoxy resin in the SPC, which enhances the adhesion of the PTFE powder to the substrate, resulting in a significant increase in the wear resistance of the coating. Figure 19b shows the change rule of rolling angle of the superhydrophobic protective coating with the number of abrasions; it can be seen that with the SPC that even after 1000 wear cycles the rolling angle is still less than 10° (7.48°), indicating it has maintained good hydrophobicity.

#### 3.3.4. Freeze-Thaw Cycle Resistance

**Figure 20:**
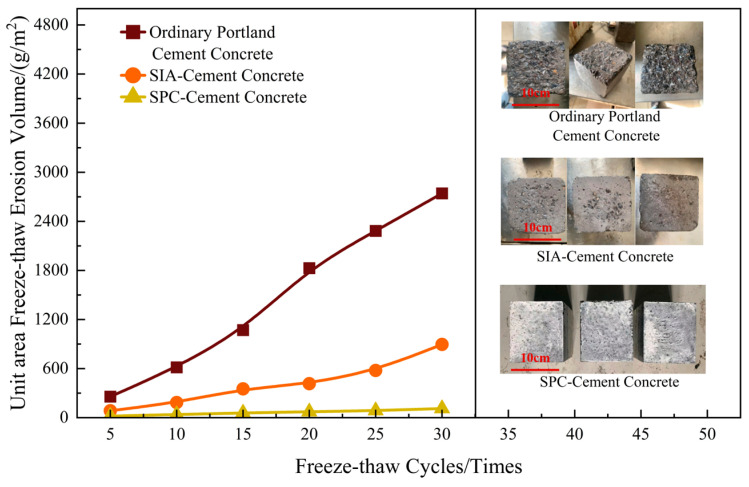
Freeze-thaw cycle test results.

Freeze-thaw cycle resistance was determined according to the test method of the freeze-thaw damage of cement concrete against de-icing solution in Specifications for Construction of Aerodrome Cement Concrete Pavement (MH 5006-2015) [37]. The erosion solution was prepared by using 97% distilled water and 3% ethylene glycol in mass ratio, the test was carried out for 30 freeze-thaw cycles, and the results are shown in Figure 20. Figure 20 shows that the amount of freeze-thaw spalling per unit surface area of all three groups of cement concrete specimens in the test increased with the increase in the number of freeze-thaw cycles. The normal cement concrete specimens without any treatment reached 2741.34 g/m^2^ of spalling per unit surface area after 30 freeze-thaw cycles. The surface area spalling of the SIA-treated cement concrete specimens was 896.21 g/m^2^ and the SPC cement concrete specimens spalled only 112.29 g/m^2^ per unit surface area. The surface spalling rate of the SPC cement concrete was reduced by 87.47% compared to the SIA. Subsequently, we tested the surface contact angle and rolling angle of the SPC after 30 freeze-thaw cycles, and the results were 152.97°and 6.46°, which indicated that the SPC still maintains good water-repellent properties after freeze-thaw.

#### 3.3.5. Slip Resistance Analysis

Slip resistance analysis was conducted by referring to the evaluation criteria in the Specifications for Pavement Evaluation and Management of Civil Airports (MH/T 5024-2019) [42], as shown in Table 4. This study measured the depth of surface structure of the cement concrete pavement sprayed with the SPC and SIA. As shown in Table 5, the average value of the SIA construction depth is 1.51 mm, which is higher than the 1.29 mm of the SPC, and the SIA performs better than the SPC in terms of roadway skid resistance. However, with reference to Table 5, the slip resistance of sprayed SPC concrete pavement is still in the “Good” grade, which meets the needs of airport pavement slip resistance.

## 4. Conclusions

1. It is difficult to achieve superhydrophobicity by relying only on the surface structure of cement concrete itself, so it is necessary to construct a rough structural layer artificially. Compared to SiC and Crn as micron substrates and HB–SiO_2_ as a nano-substrate, the combination of DIA and HL–SiO_2_ for rough structural coatings is less susceptible to cracking and creates more “air chambers” on the surface, which results in better anti-friction and hydrophobic properties.
2. The ring-opening reaction products occurring during the preparation of modified epoxy resin will seriously affect the mechanical strength of the modified epoxy resin. By controlling the reaction temperature and the ratio of reactants, the epoxy resin can be effectively controlled to be modified by dehydrated condensation, which produces more grafting products, and the content of grafted polymers is positively correlated with the hydrophobicity of the modified epoxy resin.
3. In this study, it is recommended to first spray micro- and nano-rough structure coatings (material ratio: E51:HL–SiO_2_:DIA:AE = 1:2:2:2) on the surface of cement concrete, followed by spraying a modified epoxy resin–PTFE paste (material ratio: modified–E51:PTFE:AE = 1:3:5.5) to make a superhydrophobic protective coating on the surface of cement concrete.
4. Characterised by the size of contact angle, the superhydrophobic protective coating in this study is 19.41%, 18.36%, 43.17% and 87.47% more resistant to erosion (based on HCl), UV aging, abrasion, and freeze-thaw damage to de-icer for concrete than the traditional silane surface treatment agent. The anti-slip performance is slightly lower than that of the silane surface treatment agent, but still meets the requirements of the relevant technical specifications for the use of cement concrete pavement in airports.
5. The performance test of the superhydrophobic protective coating developed in this study is limited to indoor tests, and the technology will continue to be applied in engineering construction. Meanwhile, the preparation process adopted in this study is relatively complex, and subsequently, the process still has the possibility of optimisation.

## Figures and Tables

**Table 1:** Materials and performance characteristics of superhydrophobic pavement surface coating materials.

Materials	Functionality
Natural graphite powder, octadecylamine, dopamine hydrochloride, Na_2_MoO_4_, and trimethylolaminomethane hydrochloride [21].	The coatings have the ability to actively prevent erosion from spreading, providing new insights and methods for solving erosion problems in magnesium and its alloys.
Epoxy resins, carbon nanotubes, and silica [22].	Self-repairing, can be restored to its original properties under heating conditions after damage, and it shows excellent mechanical durability and erosion resistance.
Titanium dioxide nanopowder, acetone, epoxy and polyamide resins, and stearic acid [23].	Applicable to the field of aerospace equipment, it can effectively solve the problem of aerospace equipment icing and frosting in winter and guarantee the regular operation of the equipment.
Multi-walled carbon nanotubes, graphite powder, titanium nitride, polydimethylsiloxane, and polyvinylidene fluoride [24].	The coating maintains excellent superhydrophobicity, electrical conductivity, photo-thermal properties, and electro-thermal stability in environments with large temperature variations.
KH570–modified zirconia, silica, and silicone-modified acrylic emulsions [25].	The coating has excellent abrasion resistance, sodium chloride erosion resistance, self-cleaning, and thermal stability.
Nickel, PTFE, and silicon carbide [26].	The coating is made by composite electrodeposition, which does not require additional modification with low-surface-energy substances as in the traditional preparation process, and greatly improves the erosion resistance, abrasion resistance, and durability of the coating.
A combination of modified copper mesh replica and SiO_2_ to construct micro- and nano-rough surfaces, and octadecylamine grafted humic acid as a low-surface-energy substance [27].	The copper mesh reproduces a microstructure that significantly enhances the stability of the coating.
Polyvinylidene fluoride and cerium oxide [28].	The coating is more resistant to erosion, with an erosion rate 77-times lower than that of uncoated steel and 177-times lower than that of aluminium substrates.
Silicon dioxide nanoparticles, ethyl orthosilicate, hexamethyldisilazane, and KH560 [29].	The coating has excellent superhydrophobicity, good adhesion, robustness and high light transmission.

**Table 2:** Physical and mechanical properties of cement.

Type of Cement	Setting Time/min		Flexural Strength/MPa		Compressive Strength/MPa	
	Initial Set	Final Set	3 d	28 d	3 d	28 d
P.O 42.5	172	234	5.5	8.2	31.4	50.8

**Table 3:** Infrared spectroscopy test results.

Test Number	The Stretching Vibration Absorption Peak of the Epoxy Group C–O–C	Type of Reaction
130 °C–2:1	Not found	Epoxy ring-opening reaction
130 °C–4:1	Can be found	Dehydration condensation reaction
130 °C–6:1	Can be found	Dehydration condensation reaction
140 °C–4:1	Not found	Epoxy ring-opening reaction
140 °C–6:1	Can be found	Dehydration condensation reaction
140 °C–8:1	Can be found	Dehydration condensation reaction
150 °C–6:1	Not found	Epoxy ring-opening reaction
150 °C–8:1	Can be found	Dehydration condensation reaction
150 °C–10:1	Can be found	Dehydration condensation reaction

**Table 4:** Pavement structure depth grade standards (MH/T 5024-2019) [42].

Anti-Slip Performance Grade	Good	Medium	Bad
Construction Depth	≥1.0	0.6~1.0	<0.6

**Table 5:** Test Results for Construction Depth.

Specimen Type (Chisel Treatment)	Construction Depth/mm			Construction Depth Average Value/mm
Superhydrophobic protective coating	1.29	1.31	1.27	1.29
Silane Impregnating Agent	1.53	1.49	1.52	1.51

## Data Availability

Some or all data, models, or code that support the findings of this study are available from the corresponding author upon reasonable request.

## References

[1] Qing W.S. (2022). Study on Freeze-Thaw Damage Behaviour of Airport Pavement Concrete under the Action of Deicing Fluid. Master’s Dissertation.

[2] Wang P., Chen R., Pang B., Zhang M., Sun X., Ling Z., Zhang H. (2024). Design and preparation of TiO_2_-based environmentally stable photocatalytic self-cleaning coatings. J. Compos. Mater..

[3] Song Y., Liu X. (1995). Analysis of the causes of damage to cement concrete pavement at airports in the “Three North” region. Civil Aviat. Econ. Technol..

[4] Hu S. (2003). Frost damage of cement concrete pavement and countermeasures for prevention in Northwest Gobi Beach area. Ningxia Eng. Technol..

[5] Bai K. (2009). Study on the Frost Resistance of Cement Concrete under the Action of Deicing Fluid on Airport Pavement. Master’s Dissertation.

[6] Liu W., Yuan J., Yang Q. (2016). Study on the damage mechanism of de-icing fluid on airport roadway concrete. J. East China Jiaotong Univ..

[7] Chen J., Li Y., Zheng Z., Fu Y., Cheng Q. (2014). Effect of sodium formate deicer on the frost resistance of airport runway concrete. Concr. Cem. Prod..

[8] Law K.-Y. (2015). Water–surface interactions and definitions for hydrophilicity, hydrophobicity and superhydrophobicity. Pure Appl. Chem..

[9] Pan X., Shi Z., Shi C., Ling T.-C., Li N. (2017). A review on concrete surface treatment Part I: Types and mechanisms. Constr. Build. Mater..

[10] Pan X., Shi Z., Shi C., Ling T.-C., Li N. (2017). A review on surface treatment for concrete—Part 2: Performance. Constr. Build. Mater..

[11] Zhao Y., Wang J., Hu M., Niu Y., Liang N. (2024). Fabrication of cement-based superhydrophobic coatings with enhanced self-cleaning property, chemical stability, and UV-radiation resistance. J. Build. Eng..

[12] Han S., Han T.H., Kim J.H. (2024). Surface coating method for cement-based materials to improve chloride ion penetration resistance using amine-based CO_2_ solvent. J. CO2 Util..

[13] Sun E.Y., Meng X.Z. (2024). Study on the effect of metallurgical waste on the water resistance of magnesium oxysulfate cement (MOS) coatings. Metalurgija.

[14] Xu S., Wang Q., Zhuo X., Wang N. (2024). Green Fabrication of Superhydrophobic Zeolite Coating on Cement-Based Material Surfaces to Improve Water-Resistant and Anticorrosion Properties. J. Mater. Civ. Eng..

[15] Merah A. (2021). Concrete anti-carbonation coatings: A review. J. Adhes. Sci. Technol..

[16] Agrawal A., Kumar C., Meshram A. (2021). Recovery of carbon rich material: Recycling of spent pot lining: A review. Mater. Today Proc. (P3).

[17] Yuan G., Liu Y., Ngo C.V., Guo C. (2020). Rapid fabrication of anti-corrosion and self-healing superhydrophobic aluminum surfaces through environmentally friendly femtosecond laser processing. Opt. Express.

[18] Song J., Li Y., Xu W., Liu H., Lu Y. (2019). Inexpensive and non-fluorinated superhydrophobic concrete coating for anti-icing and anti-corrosion. J. Colloid Interface Sci..

[19] Liu P., Gao Y., Wang F., Yang J., Yu X., Zhang W., Yang L. (2017). Superhydrophobic and self-cleaning behavior of Portland cement with lotus-leaf-like microstructure. J. Clean. Prod..

[20] Arabzadeh A., Ceylan H., Kim S., Gopalakrishnan K., Sassani A., Sundararajan S., Taylor P.C. (2017). Superhydrophobic Coatings on Portland Cement Concrete Surfaces. Constr. Build. Mater..

[21] Wang J. (2021). Preparation and Performance of Superhydrophobic Coating on Asphalt Concrete Surface. Master’s Dissertation.

[22] Lu L., Shao-Hua W., Hong-Ling Y., Wei-Guang G., Heng L., Zheng B.-C. (2021). Preparation and properties of hybrid silica sol/organosilicon oligomer composite transparent superhydrophobic coatings. J. East China Univ. Sci. Technol. (Nat. Sci. Ed.).

[23] Hao X., Cheng Z., Zhang Y., Xie J., Zheng H., Yue C., Sheng W. (2024). Wettability Study of an Acidified Nano-TiO_2_ Superhydrophobic Surface. ACS Omega.

[24] Xu D., Ma L., Zhang F., Wang J., Zheng K., Guo S., Chen H. (2024). Preparation of CNTs–SiO_2_ hybrids/epoxy superhydrophobic coating with self-healing property activated by shape memory effect. Compos. Commun..

[25] Jiang L., Sun J., Lin Y., Gong M., Tu K., Chen Y., Xiao T., Xiang P., Tan X. (2024). The preparation of CNTs/GP/TiN/PDMS/PVDF superhydrophobic coating with strong photothermal and electrothermal properties for antiicing and deicing. Surf. Coat. Technol..

[26] Ben J., Wu P., Wang Y., Liu J., Luo Y. (2023). Preparation and Characterization of Modified ZrO2/SiO2/Silicone-Modified Acrylic Emulsion Superhydrophobic Coating. Materials.

[27] Liu J., Fang X., Ma H., Cheng J., Xing X., Cui G., Li Z. (2023). Design and fabrication of a stable Ni–PTFE–SiC superhydrophobic anticorrosive coating by electrodeposition. NPJ Mater. Degrad..

[28] Kong X.-Q., Du H.-R., Yin S.-G., Zhang W.-J., Shen Y.-D., Fu Y. (2022). Preparation and properties of highly stable superhydrophobic cementitious material coatings. Funct. Mater..

[29] Saleh S.M., Alminderej F.M., Mohamed A.M. (2022). Superhydrophobic and Corrosion Behaviour of PVDF–CeO_2_ Composite Coatings. Materials.

[30] Woo R.S., Zhu H., Leung C.K., Kim J.K. (2008). Environmental degradation of epoxy-organoclay nanocomposites due to UV exposure: Part II residual mechanical properties. Compos. Sci. Technol..

[31] (2009). Standard Test Method for Long-Term Performance and Durability of Ordinary Concrete.

[32] (2016). National Chemical Standardization Technical Committee Chemical Reagents Sub-Technical Committee (SAC/TC 63/SC 3), General Administration of Quality Supervision, Inspection and Quarantine of the People’s Republic of China.

[33] Gong B., Ma L., Guan Q., Tan R., Wang C., Wang Z., Wang K., Liu C., Deng C., Song W. (2022). Preparation and particle size effects study of sustainable self-cleaning and durable silicon materials with superhydrophobic surface performance. J. Environ. Chem. Eng..

[34] Suo Z., Hu J.H., Jin S.S., Gong M.Y., Gong C., Li J.H. (2024). Salt-Resistant Pavement Cement Concrete Based on Ion Blocking Action and Preparation Method Thereof.

[35] Suo Z., Deng X.R., Zhang R., Zhao Z.Z., Wang X.X. (2024). Salt Erosion Resistant Asphalt Mixture Based on Ion Interference and Preparation Method.

[36] (2015). Civil Airport Pavement Field Test Procedures. Industry Standard—Civil Aviation, China.

[37] (2015). Civil Airport Pavement Field Test Procedures. Industry Standard–Civil Aviation, China.

[38] Qiu X. (2022). Research on the Material Development and Performance Test of Hydrophobic Ice–Suppressive and Anti-Skid Abrasive Layer, M.S. Master’s Dissertation.

[39] Zhang Y. (2016). Study on Seawater Environmental Response Characteristics of Polyurethane Antifouling Coatings. Ph.D. Dissertation.

[40] Xiao Y. (2012). Synthesis and Properties of Polyurethane–Modified Silicone Resin. Master’s Dissertation.

[41] Zheng Z. (2019). Preparation of Si_3N_4–GO/Solvent–Free Organosilicon Modified Epoxy Composite Coatings and Its Properties. Master’s Dissertation.

[42] (2019). Specifications for Pavement Evaluation and Management of Civil Airports.

